# Independent Long Fingers are not Essential for a Grasping Hand

**DOI:** 10.1038/srep35545

**Published:** 2016-10-19

**Authors:** Federico Montagnani, Marco Controzzi, Christian Cipriani

**Affiliations:** 1 The BioRobotics Institute|Scuola Superiore Sant’Anna, Viale R. Piaggio 34, 56025 Pontedera (PI), Italy

## Abstract

The human hand is a complex integrated system with motor and sensory components that provides individuals with high functionality and elegant behaviour. In direct connection with the brain, the hand is capable of performing countless actions ranging from fine digit manipulation to the handling of heavy objects. However the question of which movements mostly contribute to the manipulation skills of the hand, and thus should be included in prosthetic hands, is yet to be answered. Building from our previous work, and assuming that a hand with independent long fingers allowed performance comparable to a hand with coupled fingers, here we explored the actual contribution of independent fingers while performing activities of daily living using custom built orthoses. Our findings show that, when an opposable thumb is present, independent long fingers provide a measureable advantage in performing activities of daily living only when precision grasps are involved. In addition, the results suggest that the remarkable grasping skills of the human hand rely more on the independent abduction/adduction of the fingers than on their independent flexion/extension. These findings are of interest to the designers of artificial hands, including biomimetic prostheses and exoskeletons.

The development of a naturally controllable prosthesis as a substitute of the natural hand after amputation is one of the most fascinating and unsolved challenges in rehabilitation engineering and applied neuroscience. While it is well recognized that the bottleneck of current state of art prostheses is inherent to the human-machine-interface (HMI)^1^,^2^, there are also a number of limits pertaining to the physical features of the device that prevent the advent of next generation bionic hands. The most important one is certainly the lack of compact and reliable actuators with power densities similar to the human muscles, which implies that only a reduced set of movements can be fitted inside the volume of a hand^3^. In turn, the design of artificial hands requires extreme simplifications: trade-offs among desired performance, prehension capabilities and anthropomorphism are mandatory. Nevertheless the question of which movements and degrees of freedom (DoF) should be included and which ones can be ignored is a matter of debate in the communities of robotics, prosthetics and biomechanics^4^,^5^. In fact the number and the arrangement of the DoFs not only impact the prehension capabilities of the hand itself but also the extent of movements required at the proximal joints to compensate for the lack of dexterity of the hand.

The human hand is part of the upper extremity, i.e. a coordinated, multiarticular system where the combined motion of each articulation contributes to the overall function. Usually a reduced mobility in one or multiple articulations can be partially counterbalanced by means of *compensatory movements* in the other articulations. For example, individuals with transradial amputation are proficient at compensating for the missing DoFs in their hand prosthesis by changing the motions of their arms and body^6^,^7^. However, these compensatory movements (CM) often put greater forces on the anatomy and may result in residual limb pain, secondary musculoskeletal complaints and overuse syndromes^8^,^9^,^10^.

Conventional myoelectric hands, clinically available since the 70 s^11^ are relatively simple devices: a thumb opposes four fingers and all digits move simultaneously in order to form a tri-digital grip, under the action of a single actuator. In an attempt to improve such a simple design, new multi-grasp anthropomorphic hands were proposed (for a detailed review refer to our previous work^3^) and new prosthetic hands were made clinically available in the last decade^12^. Interestingly most of the work done in the area of mechatronics was aimed at enhancing the mobility of intrinsic hand movements, or in other words the number of active DoFs within the hand. As a result of these efforts the new multi-grasp prostheses (like the i-Limb by Touch Bionics) offer up to five independent digits^13^, albeit currently there are no clinically viable HMIs for controlling these digits independently^14^.

Puzzled by this paradox, in our recent study we questioned whether it is finger dexterity that is missing in current hand prostheses or instead if it is wrist dexterity the overlooked key for efficient grasping^5^. Indeed the biological wrist always contributes to the execution of a motor task involving the upper limb, thus it stands to reason to consider it as important as the hand in reaching and grasping tasks. To answer this question we compared four emulated architectures of hand-wrist prostheses using the Southampton Hand Assessment Procedure (SHAP)^15^, and we evaluated the extent of compensatory movements of unimpaired subjects wearing orthoses^5^. The results from our study confirmed our hypotheses suggesting that one single DoF added to the wrist, in particular wrist flexion/extension, accounted for the performance achievable by 22 DoFs in the hand. In addition, as observed by the experimenter, among the multitude DoFs in the hand (i.e., 22) only a few played a major role: in particular when digital manipulation was important, thumb opposition proved to be the most important movement. Thumb opposition is the ability –unique to humans– to bring in contact the fingertip of the thumb with each of the fingertips of the long fingers; it is due to two main movements one being thumb flexion/extension the other being thumb abduction/adduction.

Based on this anecdotal observation in this study we sought to further isolate the contribution of different DoFs of the human hand while grasping, by exploring the actual importance/influence of having independent long fingers while performing activities of daily living (ADLs).

To this aim we assessed and compared two emulated architectures of hands while executing ADLs, both using time-based procedures and postural/kinematics data while performing such ADLs. The hand configurations were emulated using custom built orthoses worn by ten able bodied subjects, as in our previous and other studies^5^,^16^,^17^. The configurations differed with regards to the long fingers: in one case the fingers were physically coupled and were allowed to flex/extend as single physical entity; in the other case the fingers were left unconstrained. Specifically we emulated: (i) a 3 DoFs hand (thumb flexion/extension, thumb abduction/adduction, fingers flexion/extension), and (ii) a 22 DoFs hand (the ideal hand). A third configuration resembling the conventional myoelectric hand only capable of closing/opening the digits (i.e. a 1 DoF claw) was also tested for the sake of comparison (Table 1, Fig. 1). With the aim to standardize configurations and to remove the effects of wrist flexion/extension and radial/ulnar deviation, we inhibited the latter two movements while enabling wrist pronation/supination. In fact the importance of wrist flexion/extension was already highlighted in our previous work^5^.

Akin our previous work^5^ we compared the two hand configurations using the SHAP^15^ and a Motion Analysis Test (MAT) aimed to evaluate the extent of compensatory movements in the upper extremity, as well as the kinematics data of the fingers, while performing six representative ADLs^5^. The ADLs, which gave rise to statistically different compensatory movements with the two hand configurations, were further analysed using the kinematics data of the fingers (recorded by a data glove). This supplementary analysis was performed in order to better comprehend the features that emphasize the role of independent fingers.

## Results

The ten subjects quickly learned how to master the experimental tasks using the different orthoses; the duration of the whole experiment was about 2 h for each subject, including the setup/preparation time.

### Experiment 1: SHAP

The execution times of the SHAP were used to calculate the global Index of Function (IOF) and six partial IOFs related to the six main grasp types involved in the test (spherical, power, tip, tripod, lateral, extension). The global IOF was 97 ± 0.4 [mean ± SEM (Standard Error of mean)] for configuration B (long fingers independent) and 95.5 ± 0.9 for configuration A (long fingers coupled); the t-test did not demonstrate a statistical difference between such conditions, as measured by the global IOF. Instead, as expected, in the baseline condition (Configuration C) the IOF was significantly lower, i.e., 91.3 ± 1.1 (Fig. 2). Configuration B performed better than A in five out of six partial IOFs (related to power, tip, tripod, lateral and extension grasps), albeit statistical differences between B and A were found only for tasks involving the tip (p = 0.014) and tripod (p = 0.001) grasps. In other words independent fingers (as available in configuration B) made a difference in speed compared to coupled fingers only with tasks involving the tip and tripod grasps.

### Experiment 2: MAT

Each subject executed the six tasks of the MAT in a stereotypical and repeatable manner; in fact the aggregated data from the three trials of each task demonstrated a standard deviation which was at most ~10% the mean. This occurred for the body segment movements and –when recorded– for the finger movements (Fig. 3a). The inter-subject variability was also low with regards to the body segments movements (SEM ≤ 15% the mean, across subjects); the finger movement recordings instead demonstrated substantial differences in the way each subject performed the six tasks.

Overall the compensatory movements (CMs) relative to the different joints in configuration B, fell within 10% (i.e. CM < 0.1) the Range of Motion of the joint, for most of the cases (Fig. 3 and Table S1 in Supplementary Information). The CMs associated to the shoulder elevation/depression (S_E/D_) and to the glenohumeral horizontal flexion/extension (G_F/E_) proved the largest for most of the tasks. The turning page and the carton pouring task gave rise to the largest CMs (median across joints 0.10 and 0.11, respectively) while the extension grasp to the lowest ones (median 0.02). The CMs needed for accomplishing the tasks in configuration A proved comparable to those required in configuration B, except for few specific body segments and tasks in which they demonstrated larger (Fig. 3b). Not surprisingly, the CMs required in configuration C were consistently larger than A and B (Fig. 3b).

In agreement with the results from the SHAP, the t-test on the MAT demonstrated significantly different CMs between A and B for the turning page task (primarily involving the tip grip) and the tripod grasp (one tailed paired t-test). In particular in the turning page, the CMs of the trunk rotation (T_R_; p = 0.004), of the shoulder girdle elevation/depression (S_E/D_; p = 0.006), of the glenohumeral forward-flexion/backward-extension (G_FF/BE_; p = 0.001) and horizontal flexion/extension (G_F/E_; p = 8∙10^−4^), proved significantly larger in configuration A than in B. For the tripod grasp, the CMs of the trunk lateral motion (T_L_; p = 0.02) and of both the shoulder movements, i.e. the shoulder girdle flexion/extension (S_F/E_; p = 0.005) and elevation/depression (S_E/D_; p = 0.006) were larger in configuration A than in B. Thus independent fingers promoted more natural movements than coupled fingers in two out of six tasks. In the other four tasks of the MAT no statistical differences could be observed between configurations A and B.

As they demonstrated statistically different CMs we performed a supplementary analysis on the turning page and the tripod grasp tasks, using the kinematics data of the fingers from the data-glove. In particular a linear regression analysis was performed on the data in order to assess the extent to which the angular excursions of the glove sensors covaried with each other with time. The regression analysis demonstrated variable results across subjects as highlighted by the matrixes of coefficients of determination R^2^ (Fig. 4a). The most evident common behaviour across tasks and subjects pertained the three abduction angles which proved uncorrelated with all other segments; vice-versa the MCP and PIP joints showed larger, yet scattered, inter- and intra-joint R^2^ values. In the turning page task the MCP flexion of the index finger was poorly correlated with the other joints (in all subjects except for S2 and S5). Low R^2^ values were also found for the MCP flexion versus the PIP flexions (in subjects: S1, S3, S4, S6, S9). In the tripod grasp no specific behaviours could be observed.

When compared to the other tasks the turning page and tripod grasp showed significantly smaller R^2^ values pertaining to the comparisons between MCP flexion and MCP abduction (one-way ANOVA and post-hoc pairwise comparisons using Bonferroni correction; p values in Fig. 4b caption). This result implies that grasps that have a large intra-MCP degree of correlation can be performed by hands having a single physical entity (or *virtual finger* as defined by Arbib and colleagues^18^) opposing the thumb, with performance comparable to hands having independent long fingers. Conversely, grasps with lower degree of correlation can still be performed by hands having a single virtual finger opposing the thumb but with noticeable differences compared to the normal hand.

## Discussion

In this work we sought to investigate the contribution of independent fingers during grasping by comparing the human hand performance with and without independent fingers, as measured by established standardized assessment procedures^5^,^15^,^19^,^20^,^21^,^22^. The SHAP was used because it is an objective, statistically valid method to measure hand function, very simple to use and easy to interpret^15^,^23^. The test enabled direct comparison between the configurations and allowed us to evaluate both global and single grasp type performance indexes. However, the SHAP provided a time-based metric (how fast a task is performed), without giving a direct information on how the tasks were performed. For this reason we also run a test in which we assessed the extent of non-stereotypical compensatory movements and digital mobility during ADLs using validated motion analysis techniques^5^,^19^,^20^,^21^,^22^.

The results of the SHAP proved in substantial agreement with our previous study in which unimpaired individuals (non amputees) wore an orthosis^5^. Notably, the significance of the IOFs in the different configurations, taken individually, is rather limited per se, as they represent the performance of perfectly functioning, albeit constrained, hands and limbs. Not surprisingly the IOFs, especially those achieved under configuration A and B, were not far from those achievable by the intact human hand^15^. The tasks were carried out under the control of the unimpaired sensorimotor system and thus any direct comparison of the results with those that can be expected with prosthetic hands is rather impractical. More conveniently the IOFs achieved in Configuration B could be used to form comparative results to study individuals with wrist impairments^23^. The IOFs become interesting when used to compare configurations A and B, because this comparison highlights the contribution of independent long fingers, as measured by the SHAP.

For the same reasons described above the degree of compensatory movements (CMs) required by healthy subjects wearing the orthoses are not directly comparable with those achieved by users of prostheses. Earlier studies suggested that the two groups show similar mechanisms of compensation, albeit the magnitude of compensation is greater for prosthesis users^6^,^20^. Our results indicated that – in the six investigated tasks – unimpaired individuals deprived of wrist flexion/extension and radial/ulnar deviation, compensated for the missing DoFs in ways that varied from task to task, akin to similar studies^16^,^17^ (Fig. 3 and Table S1 in Supplementary Information). It is worth to note that CMs ranging between 0.1 and 0.2 describe movements which significantly deviate (by 10–20% with respect to the ROM of the relative join) from normal execution.

When comparing the two configurations under investigation or, in other words, the influence of the long fingers, the results from the SHAP and the MAT were strongly congruent. Although configuration A allowed for just 4 DoFs while B accounted for 23, their performance did not statistically differ when the tasks primarily involved four (out of six) grasp types, in particular the spherical, the power, the lateral and the extension grasps. Conversely both the SHAP and the MAT converged on a non-trivial finding: even under perfect conditions, *even in the human hand*, independently controlled long fingers made a significant difference only with tasks involving the tip and tripod grasps. This implies that a large portion of grasps can be performed almost-naturally by a hand endowed with thumb opposition and a single virtual finger. In addition it suggests that the redundant DoFs in the human hand provide a functional advantage only for a limited subset of grasps. Notably, these grasps are infrequently used in ADLs. In their recent work Zheng and colleagues reported that tip and tripod grasps account together for 11% of total grasp instances in house maid activities and for 25% in a machinist job. If we consider the time percentage, these values drop to 8.2% (for the house maid) and to 18.7% (for the machinist)^24^. These details should be considered and properly weighed by designers of artificial hands. However, regardless their frequency in ADLs, the question is: what is the peculiarity about these grasps that makes manifest the contribution of independent fingers? The straightforward answer might sound as simple as: unlike all other grasps, the tripod and tip grasps (turning page) fall under the category of *precision grasps*, adhering, among the others, to Iberall’s and Arbib’s grasp taxonomy^25^.

In these grasps the finger pads of the thumb and of the opposing fingers are used to grasp the object. Grasp stability is ensured by means of small contact areas/pressures between the object and the finger pads. To achieve grasp stability the finger pads precisely match the geometrical configuration of the object and the fingers apply forces in the proper directions against the thumb. Hence, fingers with independent MCP flexion/extension and abduction/adduction, able to precisely oppose the thumb can provide a crucial (and measurable) advantage in these grasp types. This argument finds support from previous studies^22^,^26^ and from the linear regression analysis (Fig. 4) which showed the independency of the abduction/adduction DoFs in the tip and tripod grasps, and of the index flexion/extension in the tip grasps. Although not conclusive, our results suggest that the remarkable grasping skills of the natural hand, rely more on the independent abduction/adduction of the fingers than on their independent flexion/extension.

In the past decades roboticists have approached the grasping problem using analytical and computational approaches^4^. For example it is well known that a minimum of 3 DoFs are needed to perform basic prehension, under the assumption of a hand with rigid, hard finger, non-rolling and non-sliding contacts^27^. In this study we attempted to bring in a new dimension with a specific focus on the role of independent fingers in the human hand; our results (i) show that independent fingers provide a measureable advantage in performing ADLs only when precision grasps are involved and (ii) invite studies in which the role of abduction/adduction of the fingers is further investigated. Although our outcomes are supported only for the intact hand with perfect motor control and sensory feedback, this work provides interesting insights to engineers involved in the design of artificial hands, including prostheses and orthoses. Given the limited added value of independent fingers, their inclusion in the design of an artificial hand should be carefully assessed.

## Methods

### Participants and experimental procedure

Ten right-handed able-bodied subjects (6 males and 4 females, aged 31.2 ± 2.0 years old) free of any motor disorders participated to this study. Informed consent in accordance with the Declaration of Helsinki was obtained before conducting the experiments from each subject. This study was approved by the local ethical committee of the Scuola Superiore Sant’Anna, Pisa, Italy. The methods were carried out in accordance with the approved guidelines. The participants were asked to participate in two experiments in which they had to perform ADLs, in each of the three hand configurations. The experimental procedures were the SHAP^15^ and a motion analysis test (MAT)^5^. The MAT included a subset of tasks from the SHAP, which were representative of the six main grips used in ADLs^15^. The SHAP was performed first and the MAT later, since the MAT required a certain acquaintance with the tasks by the subjects. The three configurations were tested in a randomized order.

### Hand configurations

**Figure 1:**
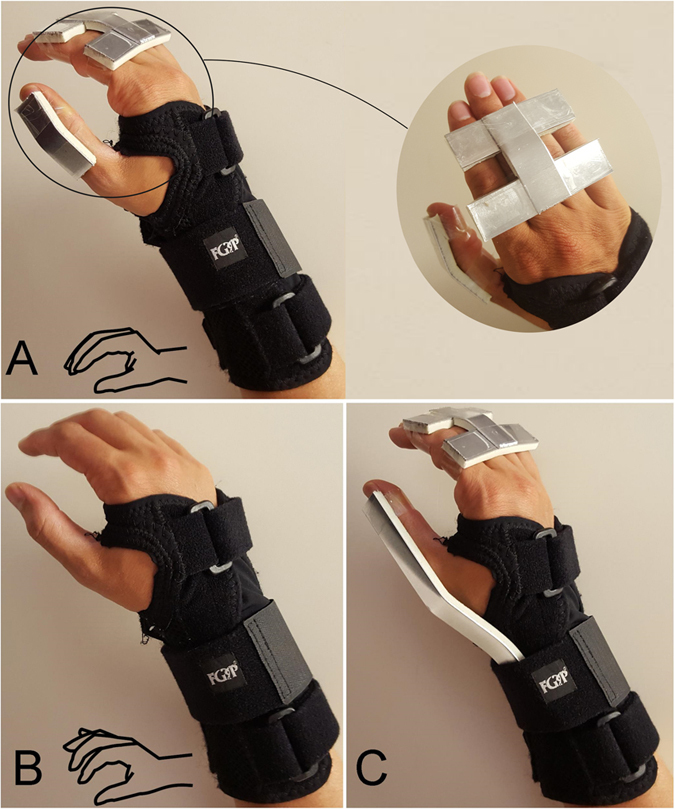
Hand configurations achieved using custom built orthoses. (**A**) 1 DoF wrist (pronation/supination) and 3 DoFs hand (open/close and thumb independent flexion/extension and abduction), the right panel depicts the aluminium frame that, together with inextensible tape, coupled the long fingers, thus impeding relative movements. (**B**) 1 DoF wrist (pronation/supination) and multi DoFs hand. (**C**) 1 DoF wrist (pronation/supination) and 1 DoF hand (open/close) (the long fingers were coupled as in configuration (**A**).

**Figure 2:**
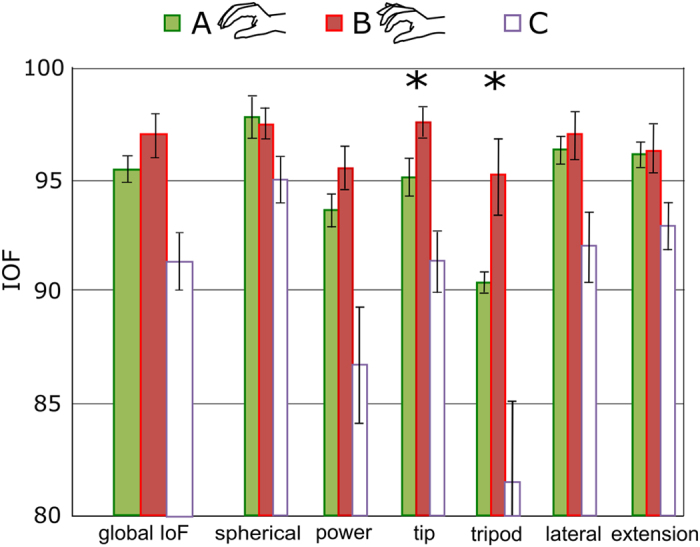
SHAP global and partial Indexes of Function. The indexes were averaged across subjects (error bars denote the standard error of mean). Asterisks denote statistical differences between configurations (A,B) (one-tailed t-test, p = 0.014 for tip grasp, p = 0.001 for tripod grasp). The data for configuration C is displayed for comparison.

**Figure 3:**
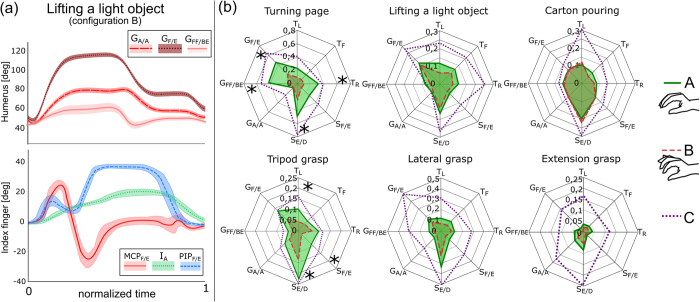
MAT. (**a**) Representative angular trajectories of the DoF of the humerus and of the index finger for one subject while “lifting a light object” in configuration B (mean values ± standard deviation). Positive values denote flexion and abduction. (**b**) Average CMs of the eight body segment angles in configuration A (continuous lines) and B (dashed lines). Asterisks denote statistical differences between configurations A and B (one tailed paired t-test; turning page: T_R_, p = 0.004; S_E/D_, p = 0.006; G_FF/BE_, p = 0.001; G_F/E_, p = 8∙10^−4^; tripod grasp: T_L_, p = 0.02; S_F/E_, p = 0.005; S_E/D_, p = 0.006). The diagrams also depict the CMs induced by configuration C (dotted line) for comparison. Acronyms: cf. Methods – Fig. 6.

**Figure 4:**
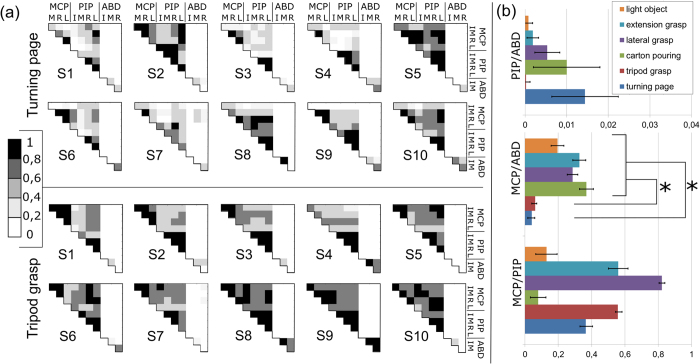
Linear regression analysis across movements of the different DoFs of the hand (configuration B) during the turning page and tripod grasp tasks. The coefficients of determination, R^2^, are displayed. All R^2^ values are statistically significant (p < 0.05). (**a**) Pairwise comparison between all DoFs recorded by the glove; each datum is the average of three trials. (**b**) Comparison of aggregated R^2^ values across tasks. Asterisks denote the cases in which the data for the turning page and tripod grasp were significantly different from the other tasks; one-way ANOVA F(5,174) = 6,24, p = 2.3∙10^−5^; post-hoc pairwise comparisons with Bonferroni correction [turning page different from light object (p = 0.034), extension grasp (p = 0.018), lateral grasp (p = 0.01) and carton pouring (p = 0.038); tripod grasp different from light object (p = 0.008), extension grasp (p = 0.007), lateral grasp (p = 0.028) and carton pouring (p = 0.008)].

Three customized orthoses were used to constrain specific DoFs of the human hand and wrist, thus to emulate different hand configurations (Table 1, Fig. 1). The configurations differed in the number and location of allowed DoFs in the hand, while the DoFs in the wrist were invariant. In particular the flexion/extension and radial/ulnar deviation were locked, whereas the pronation/supination was allowed, akin to clinically available prostheses.

#### Configuration A: 4 DoFs – long fingers coupled

This configuration emulated an improved version of a typical myoelectric prosthesis, which in addition to close/open the long fingers all together, and to pronate/supinate the wrist, is capable of opposing the thumb (i.e. two more DoFs). It was obtained using a commercial wrist orthosis (DTX 04 MANUMED manufactured by FGP S.r.l., Verona, Italy) (which inhibited its radial/ulnar deviation and flexion/extension), an aluminium frame that coupled the fingers and another splint that locked the interphalangeal joint of the thumb (Fig. 1A). Precautions were taken to ensure that the orthosis firmly coupled the fingers: to prevent relative abduction and flexion/extension among fingers and joints, the fingers were first wrapped with inextensible tape, then splinted with the aluminium frame on the dorsum (Fig. 1A). The only allowed movement was the simultaneous flexion/extension of all long fingers around the MCP joints.

#### Configuration B: 23 DoFs – long fingers independent

Conversely to configuration A, here the long fingers were all free to move (independently), as they were not splinted (Fig. 1B). The configuration mimicked a prosthetic hand with ideal motor features, capable of digital manipulation, coupled with a wrist rotator. State of art hands are far from this ideal condition, albeit some examples of advanced multi-grasp prostheses/hands were recently demonstrated^28^,^29^,^30^,^31^.

#### Configuration C: 2 DoFs – baseline condition

In this configuration the hand was splinted in a way it was only allowed to open and close as a claw while the wrist, akin to previous conditions could only pronate/supinate (Fig. 1C). This configuration, which mimicked the typical myoelectric fitting (1 DoF hand coupled with a 1 DoF wrist rotator), was included as the baseline condition.

### Experiment 1: SHAP

The ability of executing ADLs in each of the three hand configurations was evaluated using a standardized procedure for the evaluation of hand function, namely the SHAP^15^. The SHAP is divided in two parts: in the first one, composed of 12 tasks, the subject grasps and manipulates abstract objects (cylinders, tabs, spheres, etc.); in the second part he/she is required to perform 14 ADLs, such as turning a door handle, picking up coins, moving containers, etc.^15^. The SHAP is a time based protocol and the subject is required to complete each task as quickly as possible. In fact the execution times are used to calculate the global Index of Function (IOF) and six partial IOFs related to the six main grasp types involved in the test (spherical, power, tip, tripod, lateral, extension).

The subjects performed the SHAP three times, one for each configuration in a single session. The subject rehearsed each SHAP task until he/she was able to reliably perform it as specified by the SHAP assessor’s manual. The subject performed the task until satisfied that the fastest possible time was achieved. We recorded the execution times and calculated the global and partial IOFs using the equations described by Light *et al.*^15^. A one tailed paired t-test was used to compare the results between configuration A and B. A level of p < 0.05 was used as the threshold for the statistical significance.

### Experiment 2: Motion Analysis Test – MAT

The compensatory movements induced by the use of the different hand orthoses, and the actual motility of the long fingers when enabled to move independently (in configuration B), were also assessed and compared.

**Figure 5:**
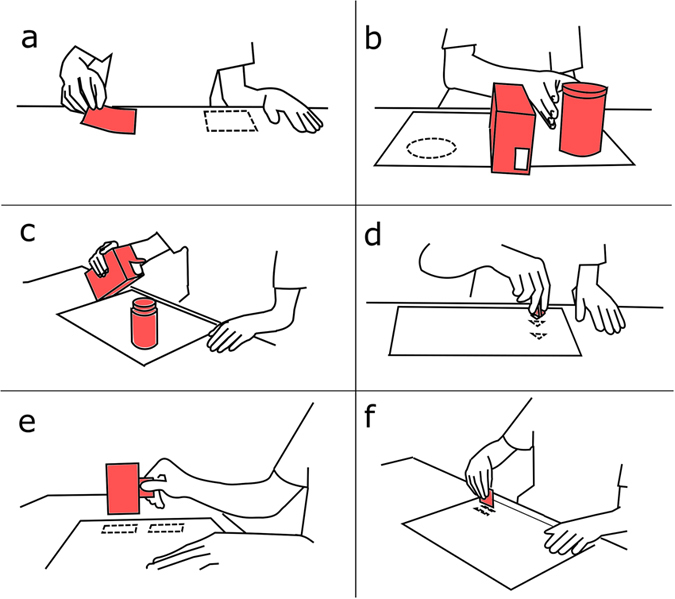
Tasks of the Motion Analysis Test. Using a subset of the SHAP materials: turning page (**a**), lifting a light object (**b**), carton pouring (**c**), tripod grasp (**d**), lateral grasp (**e**), extension grasp (**f**).

Six representative SHAP tasks, each primarily involving one of the main grip types, were chosen: turning page (tip grip), lifting a light object (a large can - spherical grip), carton pouring (power grip), tripod grasp, lateral grasp, and extension grasp (Fig. 5)^5^. Each task was performed three times under each of the three experimental configurations and in a fourth control condition, i.e., the unconstrained hand-wrist^5^,^6^,^19^,^20^. Subjects sat on a chair in front of a desk with the SHAP materials; distance from the desk and height of the chair were regulated at the beginning of the session. Subjects were asked to perform each task at self-paced speed and as naturally as possible, however, they were allowed to practice the movement before, until satisfied.

**Figure 6:**
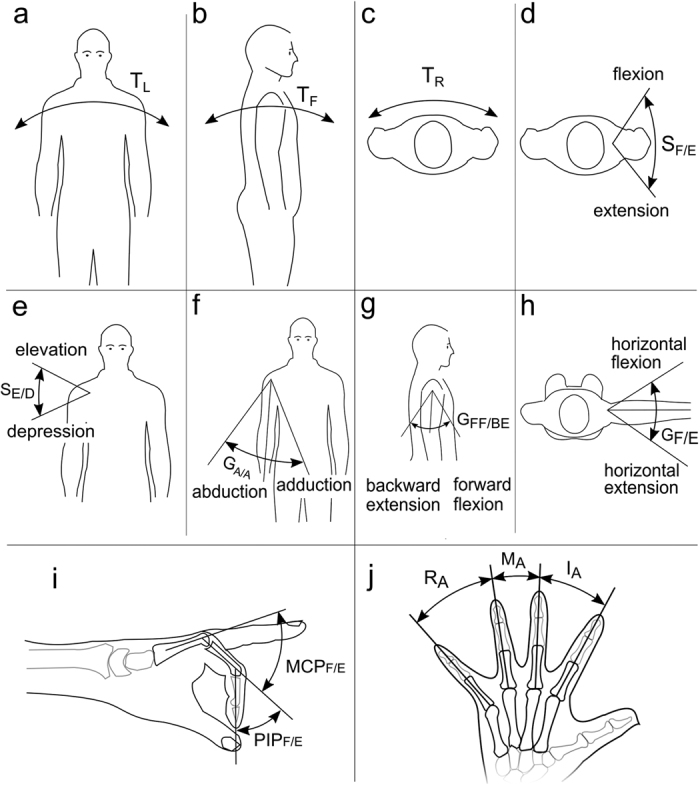
Body and hand segment angles measured during the MAT. Acronyms: T_L_ - trunk lateral motion (**a**); T_F_ - trunk forward motion (**b**); T_R_ - trunk rotation (**c**); S_F/E_ - shoulder girdle flexion/extension (**d**); S_E/D_ - shoulder girdle elevation/depression (**e**); G_A/A_ - glenohumeral abduction/adduction (**f**); G_FF/BE_ - glenohumeral forward-flexion/backward-extension (**g**); G_F/E_ - glenohumeral horizontal flexion/extension (**h**); MCP_F/E_ – metacarpophalangeal joint flexion/extension; PIP_F/E_ – proximal interphalangeal joint flexion/extension (**i**); I_A_ – index-middle fingers abduction; M_A_ – middle-ring fingers abduction; R_A_ – ring-little fingers abduction (**j**). The MCP and PIP angles were recorded for each long finger. The I_A_, M_A_ and R_A_ refer to the MCP joints abduction/adduction DoF.

A 6-cameras optoelectronic camera system (Vicon 460, by Vicon Motion Systems, Oxford, UK) and –when testing configuration B– a 18-sensor data-glove (by CyberGlove Systems LLC, San Jose, CA) were used for recording the subjects’ arm and hand kinematics (sampling rate 100 Hz). The trajectory of eight body segments was reconstructed off-line using 10 markers placed on the trunk and right arm and following the procedure described by Murgia *et al.*^19^ as in our previous and similar studies^5^,^20^. In particular the eight body segments were: the trunk lateral (T_L_), forward (T_F_) and rotation motions (T_R_) (Fig. 6a–c), the shoulder girdle flexion/extension (S_F/E_) and elevation/depression (S_E/D_) (Fig. 6d,e) the glenohumeral abduction/adduction (G_A/A_), forward-flexion/backward-extension (G_FF/BE_) and horizontal flexion/extension (G_F/E_) (Fig. 6f–h). The flexion angle of the metacarpophalangeal (MCP_F/E_) and proximal interphalangeal (PIP_F/E_) joints of the long fingers, and the angle of abduction (I_A_, M_A_, R_A_) between adjacent fingers were recorded by the data-glove (Fig. 6i,l). As in our previous study^5^ the compensatory movement pertaining to a body segment angle β, under configuration j and trial t, was computed as:


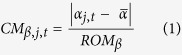


where α_j,t_ is the angular range of the body segment, 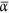 is the average range of α across the three trials in the control configuration (unconstrained hand-wrist), and ROM_β_ is the average Range of Motion of β^32^.

The differences in CMs between configurations A and B were investigated using a one tailed paired t-test, for each task of the MAT, in order to assess the effect of independent fingers (the assumptions of normality were previously verified using the Kolmogorov-Smirnov test).

The tasks that demonstrated statistically different CMs underwent a supplementary analysis using the kinematics data of the fingers from the data-glove. The duration of each trial was normalized from 0 to 1 and resampled at intervals of 0.01 of the normalized movement time. For each subject and task, the data from the three trials was first synchronized and averaged. Then, a linear regression analysis was performed on the aggregated time-series in order to assess the extent to which the angular excursions of the glove sensors covaried with each other with time. A coefficient of determination R^2^ was calculated pairwise for all the finger angles recorded (11 angles, 55 R^2^ coefficients for each task and each subject). Furthermore the R^2^ of heterogeneous angle pairs were aggregated, verified with the Kolmogorov-Smirnov test and compared across tasks using a one-way repeated measures analysis of variance (factor: tasks). Post-hoc pairwise comparisons with Bonferroni correction were performed to detect differences between tasks. A level of p < 0.05 was used as the threshold for the statistical significance, for all statistical tests.

## Additional Information

**How to cite this article**: Montagnani, F. *et al.* Independent Long Fingers are not Essential for a Grasping Hand. *Sci. Rep.* **6**, 35545; doi: 10.1038/srep35545 (2016).

## Supplementary Material

Supplementary Information

## Figures and Tables

**Table 1:** DoFs in the hand configurations.

Configuration	DoF in the wrist	DoFs in the hand	Total DoFs
A	Pronation/supination	3: Open/close fingers, thumb flexion/extension, thumb abduction/adduction	4
B	Pronation/supination	22: all DoFs in the natural hand	23
C	Pronation/supination	1: open/close all digits	2
