# Supplementary material for: Independent Long Fingers are not Essential for a Grasping Hand
Source: Sci Rep. 2016 Oct 19;6:35545. doi: 10.1038/srep35545 (PMC5069547; doi:10.1038/srep35545)
Supplement: Supplementary Information [file srep35545-s1.pdf]

Supplementary Information

Independent Long Fingers are not Essential for a Grasping Hand

Authors:

Federico Montagnani[1], Marco Controzzi[1], and Christian Cipriani[1]

configuration	body segment	Task											
		**Turning page**		**Lifting a light object**		**Carton pouring**		**Tripod grasp**		**Lateral grasp**		**Extension grasp**	
		mean	sem	mean	sem	mean	sem	mean	sem	mean	sem	mean	sem
**A**	$\mathbf{T_L}$	0,23	0,03	0,12	0,01	0,11	0,01	0,11	0,02	0,13	0,02	0,03	0,02
	$\mathbf{T_F}$	0,20	0,01	0,12	0,01	0,09	0,01	0,07	0,01	0,14	0,02	0,03	0,02
	$\mathbf{T_R}$	0,32	0,03	0,11	0,02	0,09	0,01	0,11	0,02	0,13	0,02	0,03	0,02
	$\mathbf{S_{F/E}}$	0,25	0,04	0,07	0,03	0,11	0,02	0,09	0,02	0,11	0,01	0,02	0,02
	$\mathbf{S_{E/D}}$	0,48	0,08	0,16	0,05	0,21	0,13	0,24	0,04	0,33	0,03	0,09	0,05
	$\mathbf{G_{A/A}}$	0,09	0,02	0,07	0,01	0,11	0,05	0,09	0,02	0,08	0,02	0,06	0,02
	$\mathbf{G_{FF/BE}}$	0,44	0,03	0,09	0,04	0,12	0,02	0,06	0,01	0,11	0,01	0,03	0,02
	$\mathbf{G_{F/E}}$	0,48	0,06	0,17	0,05	0,11	0,02	0,15	0,03	0,18	0,04	0,01	0,02
	**Median**	0,29		0,11		0,11		0,10		0,13		0,03	
**B**	$\mathbf{T_L}$	0,09	0,01	0,06	0,01	0,12	0,01	0,04	0,01	0,07	0,01	0,01	0,02
	$\mathbf{T_F}$	0,09	0,02	0,09	0,01	0,05	0,01	0,05	0,01	0,08	0,01	0,02	0,01
	$\mathbf{T_R}$	0,11	0,02	0,07	0,01	0,08	0,01	0,07	0,01	0,10	0,02	0,02	0,02
	$\mathbf{S_{F/E}}$	0,09	0,01	0,05	0,01	0,11	0,02	0,03	0,01	0,06	0,01	0,01	0,02
	$\mathbf{S_{E/D}}$	0,24	0,04	0,15	0,02	0,22	0,13	0,13	0,02	0,23	0,02	0,06	0,05
	$\mathbf{G_{A/A}}$	0,08	0,01	0,05	0,01	0,10	0,03	0,06	0,01	0,05	0,01	0,03	0,02
	$\mathbf{G_{FF/BE}}$	0,14	0,03	0,06	0,01	0,12	0,02	0,04	0,01	0,06	0,01	0,02	0,01
	$\mathbf{G_{F/E}}$	0,22	0,03	0,17	0,08	0,11	0,02	0,08	0,01	0,12	0,03	0,00	0,02
	**Median**	0,10		0,06		0,11		0,05		0,07		0,02	

Supplementary Table S1. Compensatory movements (CM) averaged across participants in configurations A and B. The length of the coloured bars within the cells are scaled; grey cells indicate body segments which proved statistically different between configurations. Acronyms: sem – standard error of mean; T_L - trunk lateral motion (a); T_F - trunk forward motion (b); T_R - trunk rotation (c); $S_{F/E}$ - shoulder girdle flexion/extension (d); $S_{E/D}$ - shoulder girdle elevation/depression (e); $G_{A/A}$ - glenohumeral abduction/adduction (f); $G_{FF/BE}$ - glenohumeral forward-flexion/backward-extension (g); $G_{F/E}$ - glenohumeral horizontal flexion/extension; (cf. Figure 6).
